# Ventricular fibrillation treated by an implantable cardioverter-defibrillator in antimitochondrial M2 antibody–positive myocarditis

**DOI:** 10.1016/j.hrcr.2026.07.004

**Published:** 2026-07-13

**Authors:** Kanto Saida, Atsushi Sudo, Takunori Aoyama, Hitoshi Hirano, Noboru Ichihara

**Affiliations:** Department of Cardiology, Tokyo Metropolitan Bokutoh Hospital, Tokyo, Japan

**Keywords:** Antimitochondrial M2 antibody, Myocarditis, Myositis, Implantable cardioverter-defibrillator, Ventricular fibrillation, Atrioventricular conduction disturbance, Cardiac sarcoidosis

**Key Teaching Points**

• Antimitochondrial M2 antibody-positive myositis can present predominantly with cardiac manifestations, including atrioventricular conduction disturbance, ventricular arrhythmias, and left ventricular dysfunction, even when skeletal muscle symptoms are mild.
• The phenotype may overlap with isolated cardiac sarcoidosis. Antimitochondrial M2 antibody positivity and careful skeletal muscle assessment can provide important diagnostic clues, although isolated cardiac sarcoidosis may not always be excluded completely.
• When persistent conduction disturbance requiring pacing coexists with syncope, late gadolinium enhancement, residual left ventricular dysfunction, and ventricular arrhythmias, device selection should not be reduced to routine primary-prevention criteria. An implantable cardioverter-defibrillator rather than pacemaker therapy alone may be appropriate after individualized assessment.
• Evidence guiding the optimal intensity of immunosuppression for cardiac involvement in antimitochondrial antibody-positive myositis remains limited. Antiarrhythmic therapy should also account for coexisting pauses and bradycardia.

## Introduction

Antimitochondrial antibodies, particularly antimitochondrial M2 antibodies, are best known as serologic markers of primary biliary cholangitis. However, antimitochondrial antibody–positive myositis can be accompanied by clinically important cardiac involvement, including myocarditis, left ventricular dysfunction, conduction disturbance, and ventricular arrhythmias.^1^, ^2^, ^3^, ^4^, ^5^, ^6^ Because skeletal muscle symptoms may be mild, patients can initially present to cardiology services with findings that overlap with other inflammatory cardiomyopathies, including isolated cardiac sarcoidosis. The optimal strategy for selecting an implantable cardioverter-defibrillator (ICD) rather than pacemaker therapy alone in this rare entity remains uncertain. We report a patient with antimitochondrial M2 antibody–positive myocarditis, persistent atrioventricular conduction disturbance, and ventricular ectopy who subsequently received appropriate ICD shock therapy for ventricular fibrillation (VF).

## Case report

A 61-year-old woman was admitted after syncope. She had experienced palpitations and exertional dyspnea for approximately 1 year. While watching television, she suddenly lost consciousness with upward eye deviation, limb rigidity, vomiting, and urinary incontinence; she recovered spontaneously within approximately 1 minute. She had no known previous medical history, inherited cardiomyopathy, or family history of sudden cardiac death.

On admission, her blood pressure was 128/72 mm Hg and her pulse rate was 51 beats/min (bpm). Physical examination was unrevealing. Laboratory tests showed elevated B-type natriuretic peptide (273.4 pg/mL), creatine kinase (818 U/L), creatine kinase-MB (12 U/L), and high-sensitivity troponin I (791 pg/mL); aldolase was not measured. A 12-lead electrocardiogram showed a ventricular escape rhythm with intraventricular conduction disturbance and multifocal premature ventricular complexes (PVCs). Transthoracic echocardiography demonstrated left ventricular dilation with a left ventricular end-diastolic diameter of 57 mm and a reduced left ventricular ejection fraction (LVEF) of 31%, with diffuse wall motion abnormality predominantly involving the posterior wall. Coronary angiography showed no significant obstructive coronary artery disease. Holter monitoring showed 59,658 total beats/d, a PVC burden of 29.9%, pauses of up to 3.3 seconds, and nonsustained ventricular tachycardia (VT) lasting up to 3 beats (Figure 1). Serial Holter recordings demonstrated variability in ventricular ectopy, with a PVC burden of approximately 10% on other occasions.

**Figure 1:**
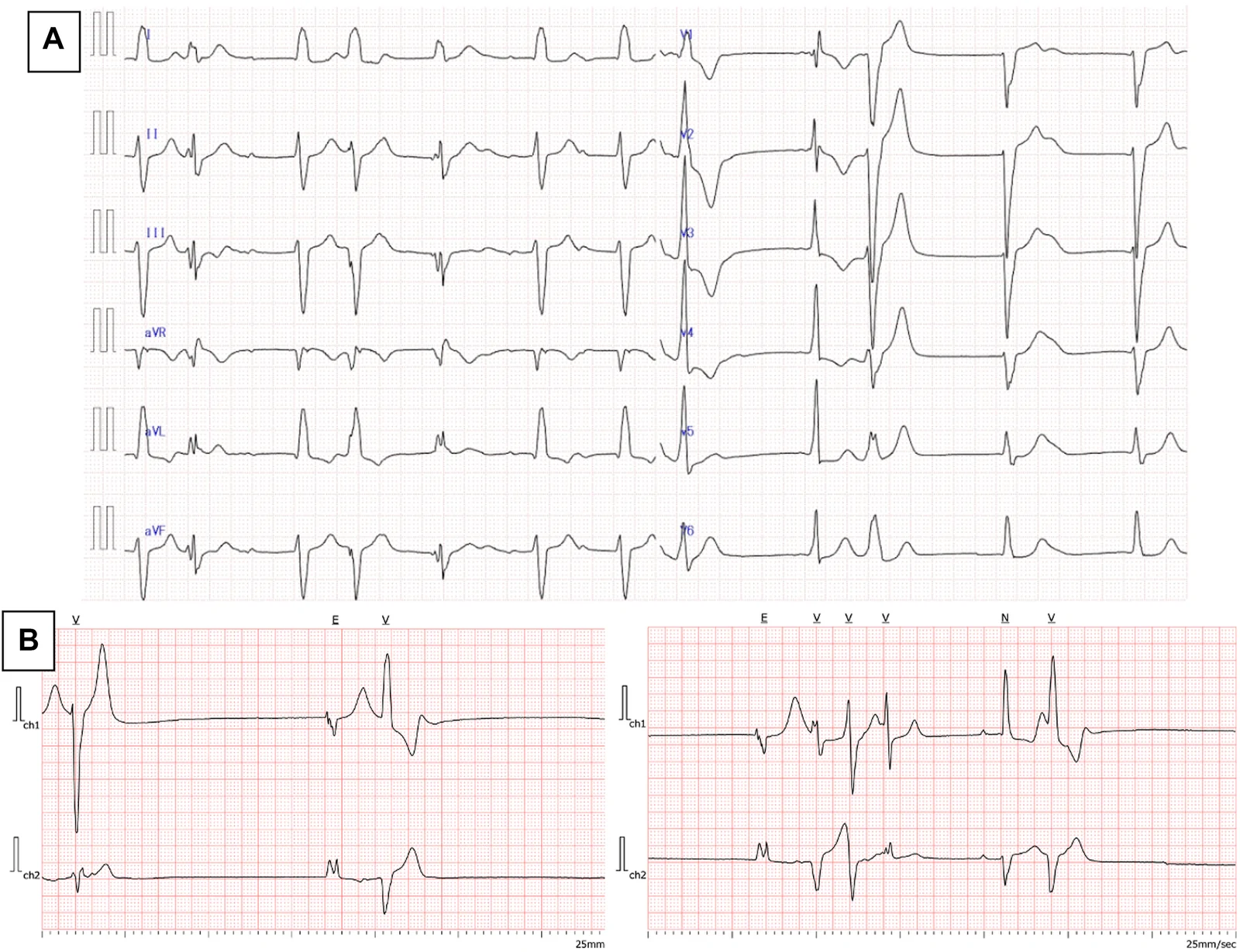
Baseline electrocardiographic and Holter findings. **A:** A 12-lead electrocardiogram showed a ventricular escape rhythm with intraventricular conduction disturbance and multifocal premature ventricular complexes. **B:** Holter recordings showed pauses of up to 3.3 seconds and nonsustained ventricular tachycardia lasting up to 3 beats.

Cardiac magnetic resonance imaging showed midwall to subepicardial late gadolinium enhancement involving the lateral to inferolateral walls and interventricular septum. T2-weighted imaging showed hyperintensity in the interventricular septum and lateral wall. T2 mapping was elevated at 62.6 ms, with lateral-wall values of 64–70 ms, whereas native T1 was within the normal range. Extracellular volume fraction and quantitative late gadolinium enhancement extent were not measured. Endomyocardial biopsy demonstrated cardiomyocyte hypertrophy, interstitial fibrosis, scattered mononuclear-cell infiltration, and histiocytes containing lipofuscin-like brown pigment (Figure 2). This pigment finding was interpreted descriptively and was not considered disease-specific, because special staining for iron or other pigment characterization was not performed. Adjacent cardiomyocyte necrosis and noncaseating epithelioid granulomas were not identified. Because adjacent cardiomyocyte necrosis was absent, the biopsy did not fulfill the Dallas criteria for active myocarditis. Immunohistochemical staining was not performed; therefore, the Marburg criteria could not be applied. Cardiac sarcoidosis was initially suspected, but contrast-enhanced computed tomography showed no hilar lymphadenopathy, and gallium scintigraphy and fluorodeoxyglucose positron emission tomography showed no abnormal cardiac uptake.

**Figure 2:**
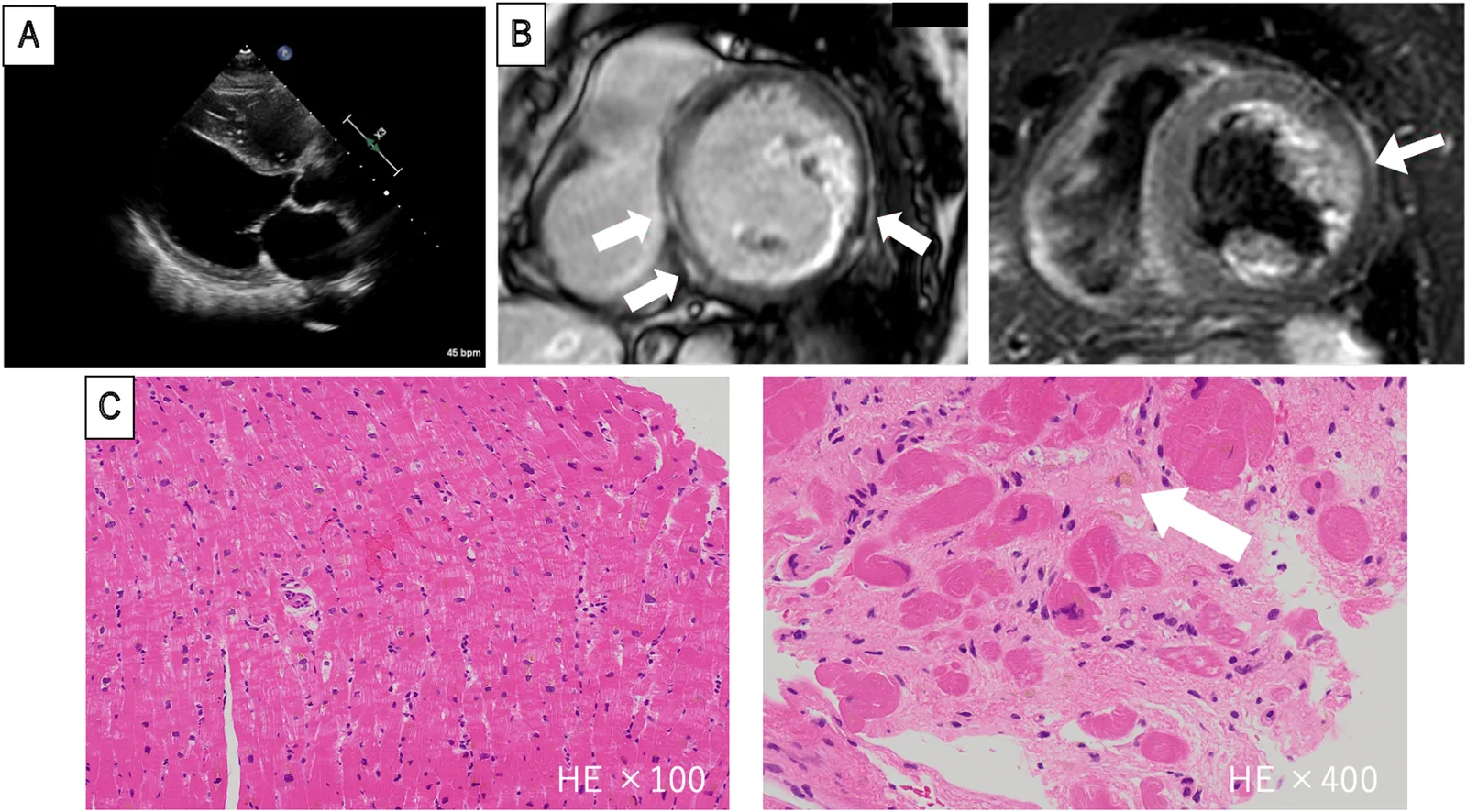
Cardiac imaging and endomyocardial biopsy. **A:** Transthoracic echocardiography showed left ventricular dilation and diffuse wall motion abnormality predominantly involving the posterior wall; the left ventricular ejection fraction was 31%. **B:** Cardiac magnetic resonance imaging showed midwall to subepicardial late gadolinium enhancement in the lateral to inferolateral walls and septum. T2-weighted imaging showed hyperintensity in the interventricular septum and lateral wall, and T2 mapping was elevated at 62.6 ms with lateral-wall values of 64–70 ms; native T1 was within the normal range. **C:** Endomyocardial biopsy showed cardiomyocyte hypertrophy, interstitial fibrosis, scattered mononuclear-cell infiltration, and histiocytes containing lipofuscin-like brown pigment (HE: left, ×100; right, ×400). *Annotated arrows* indicate mononuclear-cell infiltration and pigment-laden histiocytes. Adjacent cardiomyocyte necrosis and noncaseating epithelioid granulomas were not identified. HE = hematoxylin and eosin.

Additional immunologic testing revealed a high antinuclear antibody titer with a nuclear membrane pattern (1:640), elevated immunoglobulin M (292 mg/dL), positive antimitochondrial antibody (1:80), and markedly elevated antimitochondrial M2 antibody (533.4 U/mL; institutional reference range <7.0 U/mL). Reassessment of skeletal muscle findings revealed mild lower-limb predominant weakness. Thigh magnetic resonance imaging showed bilateral high-signal intensity on short tau inversion recovery imaging, and needle electromyography showed positive sharp waves and fasciculations at rest (Figure 3). The 2017 European League Against Rheumatism/American College of Rheumatology classification score was 6.2, corresponding to probable idiopathic inflammatory myopathy.^1^ Therefore, the patient was diagnosed with myocarditis associated with antimitochondrial M2 antibody–positive myositis.

**Figure 3:**
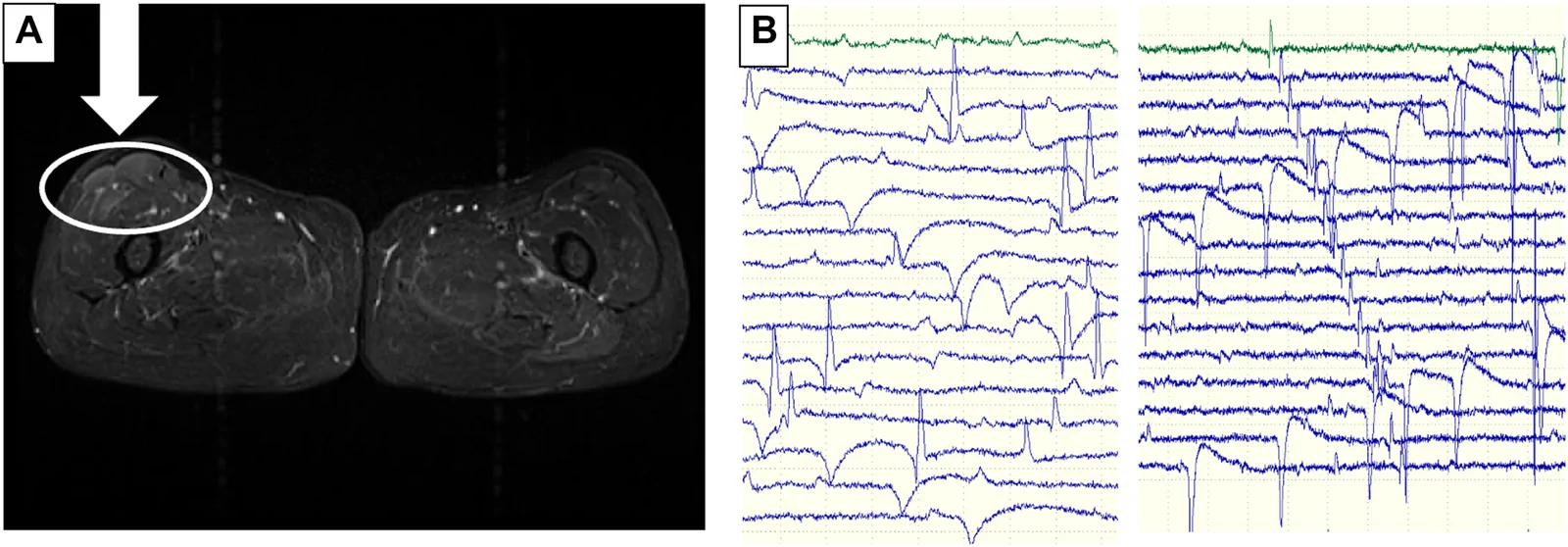
Skeletal muscle assessment. **A:** Thigh magnetic resonance imaging with short tau inversion recovery showed high-signal intensity in bilateral thigh muscles; the displayed image highlights involvement of the right lateral thigh muscles. **B:** Needle electromyography showed positive sharp waves and fasciculations at rest.

Prednisolone 1 mg/kg/d and methotrexate were initiated. Dapagliflozin and spironolactone were introduced for heart failure; renin-angiotensin system inhibitors and beta-blockers could not be adequately titrated because of hypotension and bradycardia. Atrioventricular conduction did not recover after corticosteroid initiation. Amiodarone was not started at this stage because, although suppression of ventricular ectopy was considered potentially beneficial, its bradycardic effect could have exacerbated the documented pauses.

After approximately 6 months of therapy, the LVEF had partially improved to 41%, but repeat Holter monitoring still showed pauses of up to 2.9 seconds and nonsustained VT lasting up to 6 beats. Outpatient Holter monitoring was performed approximately every 3 months. During the initial admission, sustained VT or VF was not documented during 10 days of inpatient monitoring, and the mechanism of the presenting syncope remained uncertain; therefore, immediate permanent device implantation was deferred in favor of immunosuppressive therapy and close ambulatory rhythm surveillance. In retrospect, earlier implantation of a pacing-capable defibrillator could also have been considered given the initial syncope and pauses. The Holter report became available shortly before a scheduled outpatient visit to discuss the results; however, the patient was transported emergently for recurrent syncope before that visit took place. Given the history of syncope, persistent atrioventricular conduction impairment, residual left ventricular dysfunction, extensive late gadolinium enhancement, and ventricular arrhythmias, pacemaker therapy alone was considered insufficient. A transvenous dual-chamber ICD was implanted during that hospitalization. The QRS duration was <130 ms, and ventricular ectopy was polymorphic. Cardiac resynchronization therapy defibrillator was not selected because the QRS duration was <130 ms and the high PVC burden raised concern for reduced effective biventricular pacing and cardiac resynchronization therapy nonresponse. The ICD was programmed with a monitor zone requiring 18 intervals, a VT therapy zone at 171–214 bpm requiring 16 intervals with antitachycardia pacing followed by shocks if needed, and a VF zone for rates of >214 bpm requiring 12 intervals with antitachycardia pacing during charging followed by shocks. The patient was discharged 1 week after ICD implantation. 1 week after discharge, the ICD delivered appropriate therapy for VF at 292 bpm; a 36-J shock successfully terminated the arrhythmia (Figure 4). Amiodarone was subsequently initiated, and no recurrent ICD therapy has occurred during 1 year of follow-up after the appropriate shock.

**Figure 4:**
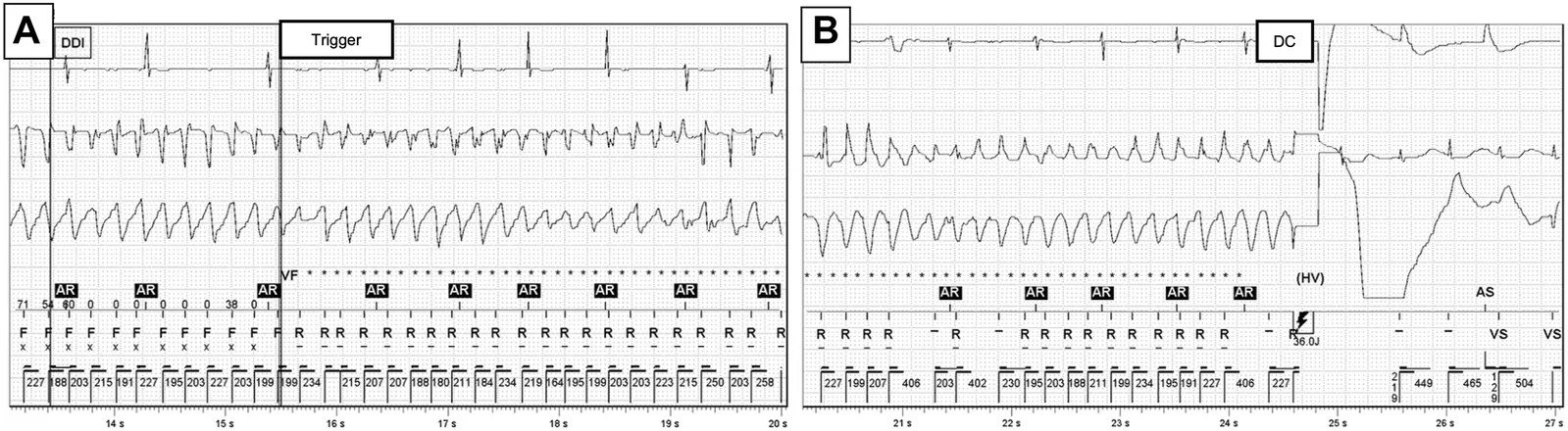
Intracardiac electrograms stored by the implantable cardioverter-defibrillator. **A:** Device interrogation documented ventricular fibrillation (VF) at 292 bpm. **B:** A 36-J shock terminated VF and restored sinus rhythm. The implantable cardioverter-defibrillator was programmed with a monitor zone requiring 18 intervals, a ventricular tachycardia therapy zone at 171–214 bpm requiring 16 intervals with antitachycardia pacing followed by shocks if needed, and a VF zone for rates of >214 bpm requiring 12 intervals with antitachycardia pacing during charging followed by shocks. bpm = beats/min.

## Discussion

This case illustrates several electrophysiological and diagnostic issues. First, antimitochondrial M2 antibody–positive myocarditis should be considered in patients with nonischemic cardiomyopathy accompanied by conduction disease and ventricular arrhythmias, even when skeletal muscle symptoms are mild. Antimitochondrial antibody–positive myositis is an uncommon inflammatory myopathy that can involve the heart and may present with conduction disease, ventricular arrhythmias, left ventricular dysfunction, or myocarditis.^2^, ^3^, ^4^, ^5^, ^6^ The phenotype can overlap with isolated cardiac sarcoidosis, particularly when conduction disease, ventricular arrhythmias, and late gadolinium enhancement coexist.^4^^,^^7^ In the present patient, the absence of hilar lymphadenopathy, negative gallium scintigraphy and fluorodeoxyglucose positron emission tomography, and absence of noncaseating epithelioid granulomas on biopsy reduced support for cardiac sarcoidosis but could not exclude an isolated cardiac phenotype. Conversely, the creatine kinase elevation, immunoglobulin M elevation, nuclear membrane antinuclear antibody pattern, antimitochondrial M2 antibody positivity, and skeletal muscle abnormalities supported antimitochondrial antibody–positive myositis with cardiac involvement.

Second, arrhythmic and bradyarrhythmic risk may persist despite immunosuppression and partial recovery of left ventricular function. In this patient, atrioventricular conduction did not recover after corticosteroid initiation, and serial Holter recordings showed persistent pauses and a variable PVC burden of approximately 10%–29.9%. A PVC-induced component may have contributed to the reduced LVEF; however, PVC-induced cardiomyopathy alone was unlikely to explain the overall phenotype, given the coexisting conduction disturbance, troponin elevation, cardiac magnetic resonance abnormalities, and biopsy evidence of myocardial injury. Steroid-responsive heart failure has been reported in this disease,^2^ but life-threatening ventricular arrhythmias may occur despite immunosuppressive therapy.^5^ In a case series, the cardiac response to immunosuppressive therapy varied, and the optimal treatment strategy remained uncertain.^6^ Escalation of immunosuppression beyond prednisolone plus methotrexate could have been considered in the present patient, particularly because conduction disturbance and arrhythmic findings persisted. However, evidence supporting a specific intensified regimen for cardiac involvement in antimitochondrial antibody–positive myositis has not been established. Further studies are needed. Amiodarone was deferred before device implantation because of concern that it might aggravate the coexisting bradyarrhythmia and pauses.

Third, standard primary-prevention ICD criteria for nonischemic cardiomyopathy cannot be directly applied to this clinical scenario. The preimplantation syncopal episodes could have been caused by bradyarrhythmia, ventricular tachyarrhythmia, or a combination of both; therefore, this case should not be framed as an unequivocal primary-prevention scenario. The clinically relevant question was whether pacemaker therapy alone would provide adequate protection in a patient with a pacing requirement and concurrent markers of ventricular arrhythmic risk. The subsequent appropriate ICD shock for VF supports the selection of an ICD rather than pacemaker therapy alone in this patient, although a single case cannot establish a general indication.

This report has limitations. It describes a single case, and the findings cannot establish optimal immunosuppressive therapy or device indications for this disease. The mechanism of the preimplantation syncope was not documented. The endomyocardial biopsy findings were nonspecific: adjacent cardiomyocyte necrosis and noncaseating epithelioid granulomas were absent, and immunohistochemical staining was not performed. The lipofuscin-like pigment within histiocytes was not further characterized by special staining and could not be used to distinguish antimitochondrial M2 antibody–positive myocarditis from other inflammatory substrates. Accordingly, the Dallas criteria for active myocarditis were not fulfilled, the Marburg criteria could not be applied, and isolated cardiac sarcoidosis could not be excluded with absolute certainty. Native T1 was within the normal range, whereas T2 mapping was elevated; extracellular volume fraction and quantitative late gadolinium enhancement extent were not measured. Genetic testing for inherited conduction disease or cardiomyopathy was not performed. Programmed ventricular stimulation for inducibility testing was not performed.

## Conclusions

Antimitochondrial M2 antibody–positive myocarditis can present primarily with conduction disturbance, ventricular arrhythmias, and left ventricular dysfunction, even when skeletal muscle symptoms are subtle. The differential diagnosis includes isolated cardiac sarcoidosis. When persistent conduction disturbance requiring pacing coexists with syncope, late gadolinium enhancement, residual ventricular dysfunction, and ventricular arrhythmias, the selection of an ICD rather than pacemaker therapy alone should be considered on an individualized basis. Evidence guiding the optimal intensity of immunosuppression in this rare condition remains limited.

## Disclosures

The authors have no conflicts of interest to disclose.
